# Associations of genetic markers of diabetes mellitus with carotid atherosclerosis: a community-based case–control study

**DOI:** 10.1186/s12933-023-01787-7

**Published:** 2023-03-09

**Authors:** Tzu-Wei Wu, Chao-Liang Chou, Chun-Fang Cheng, Shu-Xin Lu, Yih-Jer Wu, Li-Yu Wang

**Affiliations:** 1 Department of Medicine, MacKay Medical College, No. 46, Sec. 3, Zhongzheng Road, Sanzhi District, New Taipei City, Taiwan; 2 Department of Neurology, MacKay Memorial Hospital, New Taipei City, Taiwan; 3 Tamsui Health Station, Department of Health, New Taipei City Government, New Taipei City, Taiwan; 4 Institute of Biomedical Sciences, MacKay Medical College, New Taipei City, Taiwan; 5 Cardiovascular Center, Department of Internal Medicine, MacKay Memorial Hospital, Taipei, Taiwan; 6 Department of Medical Research, MacKay Memorial Hospital, Taipei, Taiwan

**Keywords:** Carotid atherosclerosis, Case–control study, Community-based study, Diabetes mellitus, FTO gene, Genetic association study, Polygenic risk score, Single nucleotide polymorphism

## Abstract

**Background:**

Diabetes mellitus (DM) is a well-established determinant of atherosclerosis and cardiovascular diseases (CVD). Recently, genome-wide association studies (GWAS) identified several single nucleotide polymorphism (SNP) significantly correlated with DM. The study aimed to explore the relationships of the top significant DM SNPs with carotid atherosclerosis (CA).

**Methods:**

We used a case–control design and randomly selected 309 cases and 439 controls with and without, respectively, carotid plaque (CP) from a community-based cohort. Eight recent GWAS on DM in East Asians reported hundreds of SNPs with genome-wide significance. The study used the top significant DM SNPs, with a p-value < 10^–16^, as the candidate genetic markers of CA. The independent effects of these DM SNPs on CA were assessed by multivariable logistic regression analyses to control the effects of conventional cardio-metabolic risk factors.

**Results:**

Multivariable analyses showed that, 9 SNPs, including rs4712524, rs1150777, rs10842993, rs2858980, rs9583907, rs1077476, rs7180016, rs4383154, and rs9937354, showed promising associations with the presence of carotid plaque (CP). Among them, rs9937354, rs10842993, rs7180016, and rs4383154 showed significantly independent effects. The means (SD) of the 9-locus genetic risk score (9-GRS) of CP-positive and -negative subjects were 9.19 (1.53) and 8.62 (1.63), respectively (p < 0.001). The corresponding values of 4-locus GRS (4-GRS) were 4.02 (0.81) and. 3.78 (0.92), respectively (p < 0.001). The multivariable-adjusted odds ratio of having CP for per 1.0 increase in 9-GRS and 4-GRS were 1.30 (95% CI 1.18–1.44; p = 4.7 × 10^–7^) and 1.47 (95% CI 1.74–9.40; p = 6.1 × 10^–5^), respectively. The means of multi-locus GRSs of DM patients were similar to those of CP-positive subjects and higher than those of CP-negative or DM-negative subjects.

**Conclusions:**

We identified 9 DM SNPs showing promising associations with CP. The multi-locus GRSs may be used as biomarkers for the identification and prediction of high-risks subjects for atherosclerosis and atherosclerotic diseases. Future studies on these specific SNPs and their associated genes may provide valuable information for the preventions of DM and atherosclerosis.

**Supplementary Information:**

The online version contains supplementary material available at 10.1186/s12933-023-01787-7.

## Background

Atherosclerosis is the gradual constriction of arteries by plaque formation within the artery walls which can reduce blood flow by > 50% [1]. Atherosclerosis is a disease of slow progression and can be aggravated by other existing conditions such as hypertension [2]. The development of atherosclerosis lesions is characterized by life-long altered lipid accumulation, especially low-density lipoprotein (LDL), and chronic inflammation in the arterial wall [1, 3]. The blood vessel endothelium then responds to blood flow turbulence and activates the recruitment of circulating immune cells. Penetrating monocytes are differentiated into macrophages which take up lipid through phagocytosis and turn into foam cells in plaques [4]. Later stages of atherosclerosis are characterized by the formation of unstable plaques which rupture and result in a local clot [5], leading to more serious clinical cardiac events such as myocardial infarction and stroke [6]. Atherosclerosis has been a leading cause of morbidity and mortality in the world especially in developed countries while the incidence of this disease is also increasing in developing countries [7]. In Taiwan, five of the top ten causes of death are related to atherosclerosis [8].

Diabetes mellitus (DM) is a group of metabolic disorders symbolized by chronic hyperglycemia leading to symptoms such as polyuria, polydipsia, and polyphagia. DM results from the low level of insulin production and/or insulin resistance of the target tissues [9]. Based on the cause of metabolic abnormalities, diabetes is mainly grouped into 2 types. Type 1 diabetes is caused by the loss of insulin-producing pancreatic β-cells due to autoimmunity, and is mostly diagnosed in children and adolescents. Type 2 diabetes accounts for about 90% of all diabetes, which begins with insulin resistance, however, the severity of the disease increase with a decline in β-cell function [10]. It is estimated that over 450 million people have diabetes worldwide and 4.2 million die because of it annually [11].

DM is a well-known risk determinant of vascular events [12]. There is a considerable increase in the literature related to DM and atherosclerosis in recent decades. Insulin resistance, which is an early preclinical stage of DM, induces several syndromes, including dyslipidemia, inflammation, hypertension, endothelial dysfunction, and vascular smooth muscle cell proliferation, which are correlated with the development of atherosclerosis [13]. Our previous community-based study also demonstrated significant relationships between DM and development and severity of carotid atherosclerosis [14]. To further explore the underlying mechanisms relating DM to atherosclerosis is scientific relevance. Recently, several genome-wide association studies (GWAS) in East Asians have identified hundreds of single nucleotide polymorphisms (SNPs) showing significant associations with DM [15–22]. It is reasonable to assume that these DM SNPs may account for the development of atherosclerosis. Therefore, we explored the relationships between the top 43 significant DM SNPs, with a p-value of < 10^–16^, and carotid atherosclerosis by a case–control study which enrolled 748 community-dwelling middle-aged adults and elders.

## Materials and methods

### Study subjects

The study used a case–control design to explore the relationships between DM SNPs and carotid atherosclerosis. The study performed stratified random sampling procedure to select study subjects from a community-based cohort, which enrolled middle-aged adults and elders from 3 townships in the northern coastal area of Taiwan [23]. From September 2010 to May 2013, a total of 1607 residents aged 40-to-74 years voluntarily provided informed consent and were enrolled. Twenty-seven subjects who lack good quality of recorded carotid ultrasound images and another 1 individual who lack blood pressure data were excluded. Another 40 subjects who had a positive history of physician-diagnosed myocardial infarction or had ever received a cardiac catheter or stent were excluded, leaving a total of 1539 subjects in the cohort.

Of the cohort members, 409 of them had detectable extracranial carotid plaques (CP). The study randomly selected 309 CP-positive individuals as the case group. The control group was a random sample of 439 individuals who had no detectable extracranial carotid plaque. The study complied with the 1975 Helsinki Declaration on ethics in medical research and were reviewed and approved by the institutional review boards of MacKay Medical College (No. P990001) and MacKay Memorial Hospital (No. 14MMHIS075).

### Measurements of anthropometric attributes and biochemical profiles

The measurements of anthropometric attributes and biochemical profiles have been described previously [23]. In brief, we used a digital system (BW-2200; NAGATA Scale Co. Ltd., Tainan, Taiwan) to measure the subject’s body weight and height. Waist circumference (WC) was measured at the level of mid-distance between the bottom of the rib cage and the top of the iliac crest. Hip circumference was the distance around the largest part of the subject’s hips. Blood pressure was measured three times, with an interval of 3 min, after 10 min of rest. The averages of repeated measurements of systolic blood pressure (SBP) and diastolic blood pressure (DBP) were used for analyses. The fasting blood levels of total cholesterol, high-density lipoprotein cholesterol (HDL-C), low-density lipoprotein cholesterol (LDL-C), triglycerides (FTG), and glucose (FPG) were determined by an autoanalyzer (Toshiba TBA c16000; Toshiba Medical System, Holliston, MA, USA) with commercial kits (Denka Seiken, Tokyo, Japan).

We also used a structured questionnaire to collect personal histories of common diseases in adults and health behaviors. In the study, hypertension was defined as subjects who had physician-diagnosed hypertension or a history of taking antihypertensive medications. Hyperlipidemia was defined as subjects having been diagnosed with high blood lipids by a physician or having a history of taking lipid-lowering medications. DM was defined as FPG ≥ 126 mg/dL or the use of insulin or other hypoglycemic agents. Cigarette smoking and alcohol drinking were defined as having smoked cigarettes or drank alcohol-containing beverages at least 4 days per week during the past month before enrollment.

### Determination of carotid plaque

The measurements of carotid atherosclerosis had been described previously [24]. In brief, we used high-resolution B-mode ultrasonography systems (GE Healthcare Vivid 7 and Vivid E9; General Electric Company, Milwaukee, USA) and followed the protocol recommended by the American Society of Echocardiography [25] to obtain the transverse and cross-sectional ultrasound images of the left and right carotid arteries. The thickness between the lumen-intima and media-adventitia interfaces was measured blindly by using automatic contouring software (GE Healthcare EchoPAC version 112.0.2; General Electric-Vingmed, Horten, Norway). In the study, a plaque was defined as a focal protrusion 50% greater than the surrounding vessel wall, an intima-media thickness (IMT) ≥ 1.5 mm, or local thickening ≥ 0.5 mm [26].

### Candidate SNPs for genetic association study

There were 8 GWAS studies on DM in East Asians [18–22] enrolled in GWAS Catalog [27]. These GWAS studies identified more than 380 SNPs with genome-wide significance [27]. This study focused on the relationships between the top 95 significant DM SNPs (with a p-value < 10^–16^) and carotid atherosclerosis (Additional file 1: Table S1). These top significant DM SNPs or their closely linked SNPs were considered for the genetic association study.

We used the Ensemble Genome Browser [28] to retrieve the linkage disequilibrium (LD) data in the 1000 Human Genome Project Phase 3-Southern Han Chinese [29]. The cut-off LD (r^2^) value of linkage was set at 0.80. Among these top 95 significant DM SNPs, 22 of them are LD with more significant SNPs, leaving a total of 73 independent DM SNPs. Among these independent DM SNPs, rs3816157, rs2844623, rs610930, rs12549902, rs13266634, and rs2383208 are the designed SNPs of the plate. Besides, another 38 SNPs of the array plate are closely linked with DM SNPs (r^2^ > 0.80). Consequently, a total of 44 SNPs were regarded as the candidate SNPs.

In the study, we used a plate (Axiom® CHB 1 Array Plate; Affymetrix Ltd, Santa Clara, CA, USA) to determine the genotypes of these 44 DM SNPs. All genotyping was performed by the National Center for Genome Medicine, Academic Sinica, Taiwan. The frequency distributions of genotypes of these 44 DM SNPs in CP-positive and -negative individuals are shown in Additional file 1: Table S2. The call rates of all typed SNPs were greater than 95% and the relative frequencies of the minor alleles of all typed SNPs were greater than 5%. Yet, SNP rs13342692 was excluded from association analysis for violation of the Hardy–Weinberg Equilibrium, leaving a total of 43 SNPs for association evaluation.

### Statistical analyses

In the study, we used the student’s t and the Pearson’s chi-square tests to compare whether there were significant differences in the anthropometric and biochemical measurements between CP-positive and -negative subjects. All anthropometric and clinical factors, which showed significant associations with carotid atherosclerosis in uni-variable analyses, were subject to multi-variable analyses. We used logistic regression model with stepwise selection method to obtain the best-fit model which includes conventional cardio-metabolic risk factors only. Then, each DM SNPs was separately added to the best-fit model to assess their independent effect on carotid atherosclerosis. The strength of association between each SNP and carotid atherosclerosis was manifested by multivariable-adjusted odds ratio (OR). To reduce the influence of false negativity, we used 0.10 as the pre-set inclusion criteria of promising SNPs. We further generated multi-locus genetic risk scores (GRSs) by summing the number of risk alleles or genotypes for each individual and then assessed the associations between GRSs and carotid atherosclerosis. All statistical analyses were performed using SAS 9.4 (SAS Institute Inc., Cary, NC, USA).

## Results

The anthropometric and clinical characteristics of CP-positive and -negative subjects are shown in Table 1. The CP-positive subjects had significantly higher means of age, BMI, waist circumference, waist-to-hip ratio, blood pressures, LDL-C, and blood glucose than the CP-negative subjects. As compared to CP-negative subjects, CP-positive subjects also had significantly higher proportions of the male sex, schooling year < 12 years, hypertension, DM, and cigarette smoking. The mean of HDL-C was significantly lower in the CP-positive subjects as compared to that of the CP-negative subjects.

**Table 1:** Comparisons of clinical characteristics between individuals with and without carotid plaque (CP)

Variables	CP-negative (n = 439)		CP-positive (n = 309)		p-values
	Mean	SD	Mean	SD	
Age at enrollment (years)	52.6	8.6	58.0	9.3	< 0.0001
Body weight (kg)	64.0	11.4	65.2	11.2	0.13
Body height (cm)	161.3	8.1	160.7	8.6	0.34
BMI (kg/m^2^)	24.5	3.6	25.2	3.4	0.011
Waist circumference (cm)	80.8	9.4	83.5	9.2	0.0001
Hip circumference (cm)	95.2	9.1	95.3	6.9	0.84
Waist-to-hip ratio (%)	84.9	6.9	87.6	7.3	< 0.0001
SBP (mm Hg)	127.2	19.0	132.7	19.4	0.0001
DBP (mm Hg)	79.3	13.3	81.3	13.7	0.050
Total cholesterol (mg/dL)	207.9	36.9	212.3	41.6	0.12
LDL-C (mg/dL)	123.8	33.2	129.3	36.5	0.035
HDL-C (mg/dL)	56.1	16.3	53.5	14.5	0.025
Fasting plasma triglyceride (mg/dL)	117.9	83.0	128.2	103.4	0.15
Fasting plasma glucose (mg/dL)	98.3	26.0	106.0	35.8	0.0015

	n	%	n	%	
Male sex	206	46.9	164	53.1	0.098
Schooling years < 12 years	288	65.6	231	74.8	0.0089
Hypertension	157	35.8	170	55	< 0.0001
Hyperlipidemia	321	73.1	233	75.4	0.48
Diabetes mellitus	39	8.9	59	19.1	< 0.0001
Cigarette smoking	65	14.8	71	23	0.0043

Multivariable logistic regression analyses of the conventional cardio-metabolic risk factors showed that the best-fit model contained age, hypertension, DM, and cigarette smoking (Table 2). The multivariable-adjusted ORs of having CP per 5.0 years increases in age was 1.38 (95% CI 1.26–1.52) and were 1.66 (95% CI 1.19–2.26), 1.60 (95% CI 1.01–2.54,), and 2.34 (95% CI 1.51–3.64) for hypertension, DM, and cigarette smoking, respectively.

**Table 2:** Association analyses for carotid atherosclerosis with baseline clinical characteristics

Variable	Univariable	Multi-variable
	OR (95% CI)	OR (95% CI)
Age (per 5.0 years)	1.39^***^ (1.28–1.52)	1.38^***^ (1.26–1.52)
Male sex	1.34^+^ (0.98–1.82)	–
BMI (per 5.0 kg/m^2^)	1.24^*^ (1.00–1.54)	–
Waist circumference (per 5.0 cm)	1.12^*^ (1.04–1.22)	–
Waist-to-hip ratio (per 5.0%)	1.24^**^ (1.10–1.38)	–
SBP (per 10.0 mm Hg)	1.08^*^ (1.00–1.18)	–
DBP (per 10.0 mm Hg)	1.08 (0.96–1.21)	–
LDL-C (per 10.0 mg/dL)	1.05^*^ (1.00–1.10)	–
HDL-C (per 10.0 mg/dL)	0.88^*^ (0.79–0.97)	–
Glucose (per 10.0 mg/dL)	1.06^*^ (1.00–1.10)	–
Schooling years < 12 years (Y/N)	1.08 (0.76–1.53)	–
Hypertension (Y/N)	1.73^**^ (1.27–2.37)	1.66^**^ (1.19–2.26)
Diabetes mellitus (Y/N)	2.42^***^ (1.57–3.74)	1.60^*^ (1.01–2.54)
Cigarette smoking (Y/N)	2.39^***^ (1.59–3.57)	2.34^***^ (1.51–3.64)

^+^0.05 < p < 0.1; ^*^0.005 < p < 0.05; ^**^0.0001 < p < 0.005; ^***^p < 0.0001

After adjustment for the effects of age, hypertension, and cigarette smoking, SNPs rs4712524, rs1150777, rs10842993, rs2858980, rs9583907, rs1077476, rs7180016, rs4383154, and rs9937354 showed promising associations with the presences of CP and were subject to further association analyses (Table 3). Model 1 showed that the multivariable-adjusted ORs of having CP was significantly elevated for rs9937354 G allele and were borderline significance for rs4712524 AA or AG genotypes, rs10842993 AG or GG genotypes, rs7180016 AA or GG genotypes, and rs4383154 AA or GG genotypes. The results of stepwise selection showed that rs10842993 AG or GG genotypes, rs7180016 AA or GG genotypes, rs4383154 AA or GG genotypes, and rs9937354 G allele were all correlated with significantly elevated ORs of having CP (Model 2).

**Table 3:** Association analyses for 9 promising SNPs with carotid atherosclerosis

Candidate SNP	Typed SNP	Risk allele (genotype)	Reference allele (genotype)	Multivariable	
				Model 1	Model 2
				OR^a^ (95% CI)	OR^b^ (95% CI)
rs4712523	rs4712524	AG or AA	GG	1.51^+^ (0.91–2.49)	–
rs4711389	rs1150777	AC	AA or CC	1.31 (0.88–1.93)	–
rs3751236	rs10842993	AG or GG	AA	1.55^+^ (0.98–2.45)	1.69^*^ (1.08–2.66)
rs7983505	rs2858980	A	G	1.23 (0.92–1.64)	–
rs9515905	rs9583907	C	T	1.23 (0.94–1.61)	–
rs4924455	rs1077476	T	G	1.20 (0.95–1.52)	–
rs8026714	rs7180016	AA or GG	AG	1.30^+^ (0.94–1.79)	1.39^*^ (1.02–1.91)
rs117267808	rs4383154	AA or GG	AG	1.68^+^ (0.99–2.84)	1.72^*^ (1.03–2.88)
rs1421085	rs9937354	G	A	1.37^*^ (1.01–1.87)	1.37^*^ (1.01–1.86)

^a^ORs for each risk allele (genotype) were adjusted for age, sex, cigarette smoking, and hypertension

^b^ORs were obtained from the best fit model contained all genetic markers and were adjusted for age, sex, cigarette smoking, and hypertension

^+^0.05 < p < 0.1; ^*^0.005 < p < 0.05; ^**^0.0001 < p < 0.005; ^***^p < 0.0001

Table 4 shows that CP-positive individuals had significantly higher mean of 4-locus GRS (4-GRS) than CP-negative individuals (4.02 ± 0.81 vs. 3.78 ± 0.92, p = 1.4 × 10^–4^). As compared with individuals who had a 4-GRS of 3 or less, the multivariable-adjusted OR of having CP for a 4-GRS of 3, 4, and 5 were 2.02 (95% CI 0.87–4.74), 2.94 (95% CI 1.29–6.69), and 4.04 (95% CI 1.74–9.40), respectively. The multivariable-adjusted OR of having CP for per 1.0 increase in 4-GRS was 1.47 (95% CI 1.22–1.77; p = 6.1 × 10^–5^). Similar results were observed for 9-locus GRS (9-GRS). The multivariable-adjusted OR of having CP for per 1.0 increase in 9-GRS was 1.30 (95% CI 1.18–1.44; p = 4.7 × 10^–7^).

**Table 4:** Association analyses for carotid atherosclerosis with multi-locus genetic risk scores (GRS)

Variable	CP-negative (n = 439)	CP-positive (n = 309)	Multivariable
	n (%)	n (%)	OR^a^ (95% CI)
9-locus GRS^b^			
Mean (SD)	8.62 (1.63)	9.19 (1.53)	
GRS			
4–6	45 (10.3)	12 (3.9)	1.00
7–8	160 (46.7)	80 (25.9)	1.91^+^ (0.92–3.97)
9–10	179 (40.8)	152 (49.2)	3.37^**^ (1.65–6.89)
≥ 11	55 (12.5)	65 (21.0)	5.59^***^ (2.56–12.19)
Per 1.0 risk score			1.30^***^ (1.18–1.44)
4-locus GRS^c^			
Mean (SD)	3.78 (0.92)	4.02 (0.81)	
GRS			
0–2	35 (8.0)	9 (2.9)	1.00
3	119 (27.1)	68 (22.0)	2.02 (0.87–4.74)
4	187 (42.6)	138 (44.7)	2.94^*^ (1.29–6.69)
5	98 (22.3)	94 (30.4)	4.04^***^ (1.74–9.40)
Per 1.0 risk score			1.47^***^ (1.22–1.77)

^a^ORs were adjusted for age, sex, cigarette smoking, and hypertension

^b^rs4712524-rs1150777-rs10842993-rs2858980-rs9583907-rs1077476-rs7180016-rs4383154-rs9937354

^c^rs10842993- rs7180016-rs4383154-rs9937354

^+^0.05 < p < 0.1; ^*^0.005 < p < 0.05; ^**^0.0001 < p < 0.005; ^***^p < 0.0001

The means (SD) of 9-GRS and 4-GRS in DM patients were 8.91 (1.53) and 4.01 (0.82), respectively. The corresponding values in individuals who had no history of DM were 8.85 (0.73) and 3.86 (0.89), respectively. Test statistics showed that DM patients had non-significantly higher means of GRSs than individuals without DM history.

## Discussion

In the present study, we performed a case–control study that enrolled 309 CP-positive subjects and 439 CP-negative subjects from a community-based cohort. Multivariable analyses of anthropometric attributes and biochemical profiles showed that DM was one of the significantly independent predictors of the best-fit regression model for the presence of CP. Among 43 tested DM SNPs, 9 of them showed promising associations with carotid atherosclerosis after controlling the effects of age, cigarette smoking, and hypertension. Although not all these promising SNPs showed significantly independent effects by multivariable analyses, there was a significantly linear trend between their composite indicator 9-GRS and the risks of carotid atherosclerosis. Four SNPs, including rs9937354, rs10842993, rs7180016, and rs4383154 showed significantly independent effects with carotid atherosclerosis. Moreover, their composite variable 4-GRS also positively linearly correlated with significantly higher ORs of having CP. Additionally, DM patients had higher means of GRSs than individuals without DM history, yet the differences were not statistically significant.

DM has been sharing many common risk factors with increased CVD risk and recent studies have shown connections under different contents [12]. DM increases the risk of ischemic stroke in the general population and in some studies DM also rises perioperative neurological complications and mortality in patients [30]. Hoke et al. followed 1065 patients with neurological asymptomatic carotid atherosclerosis as evaluated by duplex sonography prospectively during a median of 11.8 years for cause-specific mortality [31]. Multivariable regression analysis showed that the risk for all-cause and cardiovascular death of DM patients remained significantly higher even after adjustment for various established cardiovascular risk factors. In a meta-analysis of 18 studies (17,106 patients), DM was also associated with a significantly increased risk of restenosis after carotid surgical revascularization [32]. Recently, specific noncoding RNAs including microRNAs are found to be strongly associated with both DM and CVD [33, 34].

Many pathological mechanisms, including dyslipidemia with an increased level of atherogenic LDL, hyperglycemia with advanced glycation end-product formation, inflammation, and oxidative stress, have been shown to connect DM with atherosclerosis [35]. Increased levels of apolipoprotein B and atherogenic LDL were found in patients with DM [36]. Atherogenic dyslipidemia in diabetes consists of elevated serum concentrations of TG-rich lipoproteins, a high prevalence of LDL, and low concentrations of HDL [37]. Not all LDL is atherogenic and circulating LDL is the major source of lipids to be accumulated in atherosclerotic plaques. Desialylation followed by multiple enzymatic and non-enzymatic modifications results in atherogenic LDL and increases blood atherogenicity [3]. High blood glucose in DM patients can induce glycation and glycoxidation of proteins which in turn induce adhesion molecule expression in endothelium and promote the entrance of monocytes and macrophages during plaque formation. These modified proteins also promote pro-inflammatory cytokine release. Excess glycation of extracellular matrix proteins promotes their interactions with macrophages, endothelial cells, and vascular smooth muscle cells, which results in pro-inflammatory effects [38]. Atherosclerosis is currently regarded as a chronic inflammatory condition. High glucose and hyperglycemia increase neutrophil extracellular trap, a specific type of inflammatory response, which could be involved in atherosclerotic lesion formation [39, 40]. Increased ROS production and decreased antioxidant activity are known to be associated with DM and atherosclerosis development [41, 42]. In this study, DM patients had a mean 4-GRS of 4.01, which was at a clearly elevated risk stratum for carotid atherosclerosis. Our study provided an unconventional link between DM and carotid atherosclerosis, further investigation of these linking SNPs may potentially discover novel underlying mechanisms between DM and atherogenesis.

In this study, we found that rs9937354, which locates in the 1st intron of the *FTO* gene, was significantly associated with the presence of CP. We retrieved the expression data in human cells by using the Ensemble Genome Browser and found that the expression levels of FTO in multiple tissues, including heart, aorta artery, pancreas, and thyroid, are significantly correlated with rs9937354 polymorphism [43]. *FTO* gene is known to be associated with obesity and type 2 DM across diverse ethnic backgrounds [44, 45]. The FTO protein is a member of Fe(II)- and 2-oxoglutarate-dependent dioxygenases superfamily and plays a role in the demethylation of nucleic acid [46]. FTO catalyzes the demethylation of m6A to regulate the processing, maturation, and translation of the mRNAs [47]. Hepatic FTO regulates glucose and lipid metabolism and its expression is regulated by metabolic signals such as nutrients and hormones [48]. Overexpression of FTO results in increased lipid accumulation in liver and muscle cells and reduces atherogenic dyslipidemia [49]. FTO is also found to inhibit macrophage lipid influx and accelerate cholesterol efflux, which delays foam cell formation and atherosclerosis development [50].

We also observed significant associations between the presence of CP and polymorphisms of rs7180016, rs4383154, and rs10842993. SNP rs7180016 is an intron variant of the genes encoding protein regulator of cytokinesis 1 (PRC1) and PRC1-antisense 1 (PRC1-AS1). It is closely linked with 2 splice region variants (rs2301825 and rs17636091) and 1 synonymous variant (rs2301826). SNP rs7180016 also closely links with 5 3’-UTR variants and 9 noncoding transcript exon variants [51]. The expressions of PRC1 and PRC1-AS1 genes in dozens of tissues, including adipose, fat, and pancreas, are significantly correlated with rs7180016 polymorphism [51]. Recently, Ndiaye et al. found that the decreased expression of PRC1 significantly influences insulin secretion from EndoC-βH1 cells, which is an immortalized human beta cell line [52]. *PRC1* knockdown, by using *siPRC1*, significantly decreased the viability of EndoC-βH1 cells. Further network analyses showed that PRC1 was related to ‘concentration of D-glucose’, ‘quantity of insulin in blood’, ‘apoptosis of islets of Langerhans’, and ‘quantity of pancreatic cells’ [52]. More recently, Peiris et al. used an experimental approach combining *Drosophila* genetic and insulin assays with human islet genetics to explore the roles of 40 human DM genes. They identified 3 genes, including *fascetto*, *CG9650*, and *optix*, orthologs of human *PRC1*, and *BCL11A*, and *SIX3* respectively, significantly related with in vivo insulin output [53]. These in vitro and in vivo evidences indicate that PRC1 may play key roles in glucose homeostasis. Further studies are necessary to validate the roles of PRC1 in the development of atherosclerosis.

SNP rs4383154 locates in the 1st intron of glycoprotein 2 (GP2) gene. SNP rs4383154 is completely linked with a synonymous variant rs73541251 and closely linked with a missense variant rs78193826 (r^2^ = 0.945 in Southern Han Chinese) [29]. The rs78193826 T allele results in an amino acid change from valine to methionine, which may affect protein structure and function of GP2. Additionally, the expression of *GP2* gene in salivary gland, thyroid, and brain are influenced by rs4383154 polymorphism [54]. Although GP2 was identified decades ago, its’ function is still poorly understood. GP2 is predominately expressed in the pancreas and plays an antibacterial role in the gastrointestinal tract after being secreted from pancreatic acinar cells [55]. Recent meta-analysis studies showed that 3 nearby up-stream SNPs, including rs12597579, rs12597682, and rs57508503, were significantly correlated with body mass index in East Asians [56, 57]. However, these three SNPs are not closely linked with rs4383154 in Sothern Hans Chinese [29]. The role of GP2 in the pathogenesis of atherosclerotic diseases needs further exploration.

SNP rs10842993 is an intron variant of an uncategorized gene LOC105369709. It is a regulatory region variant and closely linked with 8 nearby regulatory region variants. Except for LOC105369709, Kelch-like protein 42 (*KLHL42*) gene is the closest gene to rs10842993. Additionally, the expressions of KLHL42 in multiple tissues, including arteries, blood, heart, and multiple immune cells, are significantly influenced by rs10842993 polymorphism [58]. The differential expressions of KLHL42 in multiple vascular and immune cells indicate that KLHL42 may play critical roles in immunological responses of cardiovascular system. Our speculation was supported by a recent bioinformatics study. Lu et al. analyzed the GSE100927 expression data, which contained 69 atherosclerotic carotid arteries and 35 normal carotid arteries, and identified *KLHL42* as one of the down-regulated hub genes of atherosclerosis [59]. There is no other report correlates KLHL42 with atherosclerosis and vascular events. The pathogenesis of atherosclerosis in which KLHL42 involves is needed for exploration.

## Conclusions

The study enrolled study subjects from a community-based cohort and identified critical genetic variants linking DM with atherosclerosis. Our results shed light on the mechanisms between DM and atherosclerosis development. The multi-locus GRSs showed significant association with atherosclerosis and may be used as biomarkers for the identification and prediction of high-risks subjects for atherosclerosis and atherosclerotic diseases. Future studies on the functions of these specific SNPs and their associated genes may provide more informative evidences for the prevention of DM and atherosclerosis.

## Supplementary Information

**Additional file 1: Table S1.** Information of the top 95 significant DM genetic markers. **Table S2. **Genotype distributions of 44 DM SNPs in CP-positive and -negative subjects.

## Data Availability

The datasets used and/or analysed during the current study are available from the corresponding author on reasonable request.

## Abbreviations

BMI: Body mass index
CVD: Cardiovascular diseases
DBP: Diastolic blood pressure
DM: Diabetes mellitus
FPG: Fasting plasma glucose
FTG: Fasting triglycerides
FTO: Fat mass and obesity associated
HDL-C: High-density lipoprotein cholesterol
IMT: Intima-media thickness
LDL-C: Low-density lipoprotein cholesterol
SBP: Systolic blood pressure
SNP: Single nucleotide polymorphism
WC: Waist circumference

## References

[1] Björkegren JLM, Lusis AJ (2022). Atherosclerosis: recent developments. Cell.

[2] Vasdev S, Gill V, Singal P (2007). Role of advanced glycation end products in hypertension and atherosclerosis: therapeutic implications. Cell Biochem Biophys.

[3] Summerhill VI, Grechko AV, Yet SF, Sobenin IA, Orekhov AN (2019). The atherogenic role of circulating modified lipids in atherosclerosis. Int J Mol Sci.

[4] Cheng F, Torzewski M, Degreif A, Rossmann H, Canisius A, Lackner KJ (2013). Impact of glutathione peroxidase-1 deficiency on macrophage foam cell formation and proliferation: implications for atherogenesis. PLoS ONE.

[5] Galis ZS, Sukhova GK, Lark MW, Libby P (1994). Increased expression of matrix metalloproteinases and matrix degrading activity in vulnerable regions of human atherosclerotic plaques. J Clin Invest.

[6] Robinson JG, Fox KM, Bullano MF, Grandy S, SHIELD Study Group (2009). Atherosclerosis profile and incidence of cardiovascular events: a population- based survey. BMC Cardiovasc Disord.

[7] Roth GA, Mensah GA, Johnson CO, Addolorato G, Ammirati E, Baddour LM (2020). Global burden of cardiovascular diseases and risk factors, 1990–2019: update from the GBD 2019 study. J Am Coll Cardiol.

[8] Li YH, Ueng KC, Jeng JS, Charng MJ, Lin TH, Chien KL (2017). 2017 Taiwan lipid guidelines for high risk patients. J Formos Med Assoc.

[9] Kharroubi AT, Darwish HM (2015). Diabetes mellitus: the epidemic of the century. World J Diabetes.

[10] Stancáková A, Javorský M, Kuulasmaa T, Haffner SM, Kuusisto J, Laakso M (2009). Changes in insulin sensitivity and insulin release in relation to glycemia and glucose tolerance in 6,414 Finnish men. Diabetes.

[11] Saeedi P, Petersohn I, Salpea P, Malanda B, Karuranga S, Unwin N (2019). Global and regional diabetes prevalence estimates for 2019 and projections for 2030 and 2045: results from the International Diabetes Federation Diabetes Atlas, 9th edition. Diabetes Res Clin Pract.

[12] Cosentino F, Grant PJ, Aboyans V, Bailey CJ, Ceriello A, Delgado V (2020). 2019 ESC Guidelines on diabetes, pre-diabetes, and cardiovascular diseases developed in collaboration with the EASD. Eur Heart J.

[13] Di Pino A, DeFronzo RA (2019). Insulin resistance and atherosclerosis: implications for insulin-sensitizing agents. Endocr Rev.

[14] Wu TW, Chou CL, Cheng CF, Lu SX, Wang LY (2022). Prevalences of diabetes mellitus and carotid atherosclerosis and their relationships in middle-aged adults and elders: a community-based study. J Formos Med Assoc.

[15] Yasuda K, Miyake K, Horikawa Y, Hara K, Osawa H, Furuta H (2008). Variants in *KCNQ1* are associated with susceptibility to type 2 diabetes mellitus. Nat Genet.

[16] Takeuchi F, Serizawa M, Yamamoto K, Fujisawa T, Nakashima E, Ohnaka K (2009). Confirmation of multiple risk loci and genetic impacts by a genome-wide association study of type 2 diabetes in the Japanese population. Diabetes.

[17] Cui B, Zhu X, Xu M, Guo T, Zhu D, Chen G (2011). A genome-wide association study confirms previously reported loci for type 2 diabetes in Han Chinese. PLoS ONE.

[18] Hara K, Fujita H, Johnson TA, Yamauchi T, Yasuda K, Horikoshi M (2014). Genome-wide association study identifies three novel loci for type 2 diabetes. Hum Mol Genet.

[19] Imamura M, Takahashi A, Yamauchi T, Hara K, Yasuda K, Grarup N (2016). Genome-wide association studies in the Japanese population identify seven novel loci for type 2 diabetes. Nat Commun.

[20] Suzuki K, Akiyama M, Ishigaki K, Kanai M, Hosoe J, Shojima N (2019). Identification of 28 new susceptibility loci for type 2 diabetes in the Japanese population. Nat Genet.

[21] Ishigaki K, Akiyama M, Kanai M, Takahashi A, Kawakami E, Sugishita H (2020). Large-scale genome-wide association study in a Japanese population identifies novel susceptibility loci across different diseases. Nat Genet.

[22] Spracklen CN, Horikoshi M, Kim YJ, Lin K, Bragg F, Moon S (2020). Identification of type 2 diabetes loci in 433,540 East Asian individuals. Nature.

[23] Wu TW, Hung CL, Liu CC, Wu YJ, Wang LY, Yeh HI (2017). Associations of cardiovascular risk factors with carotid intima-media thickness in middle-age adults and elders. J Atheroscler Thromb.

[24] Chou CL, Wu YJ, Hung CL, Liu CC, Wang SD, Wu TW (2019). Segment-specific prevalence of carotid artery plaque and stenosis in middle-aged adults and elders in Taiwan: a community-based study. J Formos Med Assoc.

[25] Stein JH, Korcarz CE, Hurst RT, Lonn E, Kendall CB, Mohler ER (2008). Use of carotid ultrasound to identify subclinical vascular disease and evaluate cardiovascular disease risk: a consensus statement from the American Society of Echocardiography Carotid Intima-Media Thickness Task Force. Endorsed by the Society for Vascular Medicine. J Am Soc Echocardiogr.

[26] Touboul PJ, Hennerici MG, Meairs S, Adams H, Amarenco P, Bornstein N, et al. Mannheim carotid intima-media thickness and plaque consensus (2004–2006–2011). An update on behalf of the advisory board of the 3rd, 4th and 5th watching the risk symposia, at the 13th, 15th and 20th European Stroke Conferences, Mannheim, Germany, 2004, Brussels, Belgium, 2006, Hamburg, Germany, 2011. Cerebrovasc Dis 2012;34:290–6. 10.1159/000343145 PMC3760791 23128470

[27] Buniello A, MacArthur JAL, Cerezo M, Harris LW, Hayhurst J, Malangone C (2019). The NHGRI-EBI GWAS Catalog of published genome-wide association studies, targeted arrays and summary statistics 2019. Nucleic Acids Res.

[28] Cunningham F, Allen JE, Allen J, Alvarez-Jarreta J, Amode MR, Armean IM (2022). Ensembl 2022. Nucleic Acids Res.

[29] Yates A, Akanni W, Amode MR, Barrell D, Billis K, Carvalho-Silva D (2016). Ensembl 2016. Nucleic Acids Res.

[30] Dimic A, Markovic M, Vasic D, Dragas M, Zlatanovic P, Mitrovic A (2019). Impact of diabetes mellitus on early outcome of carotid endarterectomy. Vasa.

[31] Hoke M, Schillinger M, Minar E, Goliasch G, Binder CJ, Mayer FJ (2019). Carotid ultrasound investigation as a prognostic tool for patients with diabetes mellitus. Cardiovasc Diabetol.

[32] Texakalidis P, Tzoumas A, Giannopoulos S, Jonnalagadda AK, Jabbour P, Rangel-Castilla L (2019). Risk factors for restenosis after carotid revascularization: a meta-analysis of hazard ratios. World Neurosurg.

[33] Mahjoob G, Ahmadi Y, Fatima Rajani H, Khanbabaei N, Abolhasani S (2022). Circulating microRNAs as predictive biomarkers of coronary artery diseases in type 2 diabetes patients. J Clin Lab Anal.

[34] Tang N, Jiang S, Yang Y, Liu S, Ponnusamy M, Xin H, Yu T (2018). Noncoding RNAs as therapeutic targets in atherosclerosis with diabetes mellitus. Cardiovasc Ther.

[35] Poznyak A, Grechko AV, Poggio P, Myasoedova VA, Alfieri V, Orekhov AN (2020). The diabetes mellitus-atherosclerosis connection: the role of lipid and glucose metabolism and chronic inflammation. Int J Mol Sci.

[36] Albers JJ, Marcovina SM, Imperatore G, Snively BM, Stafford J, Fujimoto WY (2008). Prevalence and determinants of elevated apolipoprotein B and dense low-density lipoprotein in youths with type 1 and type 2 diabetes. J Clin Endocrinol Metab.

[37] Hirano T (2018). Pathophysiology of diabetic dyslipidemia. J Atheroscler Thromb.

[38] Katakami N (2018). Mechanism of development of atherosclerosis and cardiovascular disease in diabetes mellitus. J Atheroscler Thromb.

[39] Menegazzo L, Ciciliot S, Poncina N, Mazzucato M, Persano M, Bonora B (2015). NETosis is induced by high glucose and associated with type 2 diabetes. Acta Diabetol.

[40] Nahrendorf M, Swirski FK (2015). Immunology. Neutrophil-macrophage communication in inflammation and atherosclerosis. Science.

[41] Chew P, Yuen DY, Stefanovic N, Pete J, Coughlan MT, Jandeleit-Dahm KA (2010). Antiatherosclerotic and renoprotective effects of ebselen in the diabetic apolipoprotein E/GPx1-double knockout mouse. Diabetes.

[42] Di Marco E, Jha JC, Sharma A, Wilkinson-Berka JL, Jandeleit-Dahm KA, de Haan JB (2015). Are reactive oxygen species still the basis for diabetic complications?. Clin Sci.

[43] Website https://www.ensembl.org/Homo_sapiens/Variation/Mappings?db=core;r=16:53765435-53766435; v=rs9937354;vdb=variation;vf=730580740; Accessed on 15 Nov 2022.

[44] Frayling TM, Timpson NJ, Weedon MN, Zeggini E, Freathy RM, Lindgren CM (2007). A common variant in the *FTO* gene is associated with body mass index and predisposes to childhood and adult obesity. Science.

[45] Yeo GS (2014). The role of the FTO (Fat Mass and Obesity Related) locus in regulating body size and composition. Mol Cell Endocrinol.

[46] Gerken T, Girard CA, Tung YC, Webby CJ, Saudek V, Hewitson KS (2007). The obesity-associated *FTO* gene encodes a 2-oxoglutarate-dependent nucleic acid demethylase. Science.

[47] Fu Y, Jia G, Pang X, Wang RN, Wang X, Li CJ (2013). FTO-mediated formation of N6-hydroxymethyladenosine and N6-formyladenosine in mammalian RNA. Nat Commun.

[48] Mizuno TM (2018). Fat Mass and Obesity Associated (FTO) gene and hepatic glucose and lipid metabolism. Nutrients.

[49] Kang H, Zhang Z, Yu L, Li Y, Liang M, Zhou L (2018). FTO reduces mitochondria and promotes hepatic fat accumulation through RNA demethylation. J Cell Biochem.

[50] Mo C, Yang M, Han X, Li J, Gao G, Tai H (2017). Fat mass and obesity-associated protein attenuates lipid accumulation in macrophage foam cells and alleviates atherosclerosis in apolipoprotein E-deficient mice. J Hypertens.

[51] Website https://www.ensembl.org/Homo_sapiens/Variation/Explore?r=15:90982545-90983545;v=rs7180016;vdb=variation;vf=105439139; Accessed on 15 Nov 2022

[52] Ndiaye FK, Ortalli A, Canouil M, Huyvaert M, Salazar-Cardozo C, Lecoeur C (2017). Expression and functional assessment of candidate type 2 diabetes susceptibility genes identify four new genes contributing to human insulin secretion. Mol Metab.

[53] Peiris H, Park S, Louis S, Gu X, Lam JY, Asplund O (2018). Discovering human diabetes-risk gene function with genetics and physiological assays. Nat Commun.

[54] Website https://www.ensembl.org/Homo_sapiens/Variation/Explore?r=16:20316844-20317844;v=rs78193826;vdb=variation;vf=733841797; Accessed on 15 Nov 2022.

[55] Lin Y, Nakatochi M, Sasahira N, Ueno M, Egawa N, Adachi Y (2021). Glycoprotein 2 in health and disease: lifting the veil. Genes Environ.

[56] Wen W, Cho YS, Zheng W, Dorajoo R, Kato N, Qi L (2012). Meta-analysis identifies common variants associated with body mass index in east Asians. Nat Genet.

[57] Sakaue S, Kanai M, Tanigawa Y, Karjalainen J, Kurki M, Koshiba S (2021). A cross-population atlas of genetic associations for 220 human phenotypes. Nat Genet.

[58] Website https://www.ensembl.org/Homo_sapiens/Variation/Explore?r=12:27811215-27812215;v=rs10842993;vdb=variation;vf=731358096; Accessed on 15 Nov 2022.

[59] Lu Y, Zhang X, Hu W, Yang Q (2021). The identification of candidate biomarkers and pathways in atherosclerosis by integrated bioinformatics analysis. Comput Math Methods Med.

