# Supplementary material for: Associations of genetic markers of diabetes mellitus with carotid atherosclerosis: a community-based case–control study
Source: Cardiovasc Diabetol. 2023 Mar 9;22:51. doi: 10.1186/s12933-023-01787-7 (PMC9999522; doi:10.1186/s12933-023-01787-7)
Supplement: Supplementary file 1 — Additional file 1: Table S1. Information of the top 95 significant DM genetic markers. Table S2. Genotype distributions of 44 DM SNPs in CP-positive and -negative subjects. [file 12933_2023_1787_MOESM1_ESM.docx]

**Table S1. Information of the top 95 significant DM genetic markers**

| SNP | Chr | POS (GRCh38) | Mapped_gene | Study accession | Type of variant | Type of SNP |
| --- | --- | --- | --- | --- | --- | --- |
| rs10634531 | 2 | 632790 | *TMEM18, AC093326.1* | GCST010118 | Intergeic | Independent SNP |
| rs1260326 | 2 | 27508073 | *GCKR* | GCST010118 | Missense | Independent SNP |
| rs11926494 | 3 | 23217123 | *UBE2E2* | GCST010118 | Intronic | Independent SNP |
| rs12633613 | 3 | 23349016 | *UBE2E2* | GCST90013693 | Intronic | Independent SNP |
| rs67114627 | 3 | 63919040 | *ATXN7* | GCST010118 | Intronic | Independent SNP |
| rs3816157 | 3 | 64013646 | *PSMD6-AS1, PSMD6* | GCST90013693 | NCTEV | Independent SNP |
| rs13092876 | 3 | 185777532 | *IGF2BP2* | GCST010118 | Intronic | Tagged SNP |
| rs4686392 | 3 | 185806293 | *IGF2BP2* | GCST90013693 | Intronic | LD with rs13092876 |
| rs1470579 | 3 | 185811292 | *IGF2BP2* | GCST003400 | Intronic | LD with rs13092876 |
| rs730831 | 4 | 1246511 | *CTBP1* | GCST90013693 | Intronic | LD with rs7656416 |
| rs79407053 | 4 | 1250430 | *CTBP1-DT* | GCST007847 | NCTEV | LD with rs7656416 |
| rs7656416 | 4 | 1260747 | *CTBP1-DT* | GCST010118 | Intronic | Tagged SNP |
| rs10011838 | 4 | 152599127 | *RPS3AP18, AC023424.1* | GCST010118 | 3’-UTR | Independent SNP |
| rs459193 | 5 | 56510924 | *RPL26P19, C5orf67* | GCST90013693 | Intergeic | LD with rs256904 |
| rs256904 | 5 | 56514478 | *C5orf67* | GCST010118 | Intronic | Tagged SNP |
| rs4712523 | 6 | 20657333 | *CDKAL1* | GCST000383 | Intronic | Independent SNP |
| rs35612982 | 6 | 20682391 | *CDKAL1* | GCST003400 | Intronic | LD with rs9350271 |
| rs9350271 | 6 | 20682933 | *CDKAL1* | GCST010118 | Intronic | Tagged SNP |
| rs10440833 | 6 | 20687890 | *CDKAL1* | GCST007847 | Intronic | LD with rs9350271 |
| rs76541615 | 6 | 31058459 | *HCG22* | GCST010118 | Intronic | Independent SNP |
| rs2844623 | 6 | 31264766 | *AL662844.2, HLA-C* | GCST007847 | Intergeic | Independent SNP |
| rs4711389 | 6 | 34246893 | *SMIM29* | GCST010118 | NCTEV | Independent SNP |
| rs9380826 | 6 | 39069886 | *GLP1R* | GCST90013693 | Intronic | Independent SNP |
| rs742762 | 6 | 39078868 | *GLP1R* | GCST010118 | Intronic | Independent SNP |
| rs6918311 | 6 | 136966564 | *AL365223.1, RPL35AP3* | GCST90013693 | Intergeic | Independent SNP |
| rs17168486 | 7 | 14858657 | *DGKB* | GCST010118 | Intronic | Independent SNP |
| rs610930 | 7 | 69724740 | *AUTS2* | GCST010118 | Intronic | Independent SNP |
| rs10618080 | 7 | 70197262 | *AUTS2* | GCST90013693 | Intronic | Independent SNP |
| rs12698877 | 7 | 70231919 | *AUTS2* | GCST010118 | Intronic | Independent SNP |
| rs117737118 | 7 | 126886937 | *GRM8* | GCST010118 | Intronic | Independent SNP |
| rs2233580 | 7 | 127613496 | *PAX4* | GCST010118 | Missense | Independent SNP |
| rs61342118 | 7 | 128121865 | *MIR129-1, SND1* | GCST010118 | Intergeic | Independent SNP |
| rs12549902 | 8 | 41651740 | *ANK1, NKX6-3* | GCST90013693 | Intergeic | Independent SNP |
| rs33981001 | 8 | 41655130 | *ANK1* | GCST010118 | Intronic | Independent SNP |
| rs13266634 | 8 | 117172544 | *SLC30A8* | GCST010118 | Missense | Independent SNP |
| rs4237150 | 9 | 4290085 | *GLIS3* | GCST010118 | Intronic | Independent SNP |
| rs2383208 | 9 | 22132077 | *CDKN2B-AS1, AL157937.1* | GCST003400 | Intergeic | Independent SNP |
| rs10965247 | 9 | 22132730 | *AL157937.1, CDKN2B-AS1* | GCST90013693 | Intergeic | LD with rs10965248 |
| rs10965248 | 9 | 22132879 | *AL157937.1, CDKN2B-AS1* | GCST010118 | Intergeic | Tagged SNP |
| rs10811661 | 9 | 22134095 | *AL157937.1, CDKN2B-AS1* | GCST002128 | Intergeic | LD with rs10965248 |
| rs2796441 | 9 | 81694033 | *AL591368.1* | GCST010118 | Intronic | Independent SNP |
| rs28624681 | 9 | 136343450 | *GPSM1* | GCST007847 | Intronic | Independent SNP |
| rs28445975 | 9 | 136349924 | *GPSM1* | GCST90013693 | Intronic | Independent SNP |
| rs376993806 | 9 | 136352136 | *GPSM1* | GCST010118 | Intronic | Independent SNP |
| rs10795945 | 10 | 12260608 | *RN7SL198P, CDC123* | GCST007847 | Intergeic | LD with rs11257657 |
| rs4747971 | 10 | 12261372 | *RN7SL198P, CDC123* | GCST90013693 | Intergeic | LD with rs11257657 |
| rs11257657 | 10 | 12267140 | *RN7SL198P, CDC123* | GCST010118 | Intergeic | Tagged SNP |
| rs34204798 | 10 | 79191374 | *ZMIZ1* | GCST010118 | Intronic | Independent SNP |
| rs147689733 | 10 | 91832946 | *TNKS2* | GCST010118 | Intronic | Independent SNP |
| rs35906730 | 10 | 92675916 | *HHEX* | GCST010118 | Intergeic | Independent SNP |
| rs12219514 | 10 | 92706682 | *HHEX* | GCST007847 | Intergeic | Independent SNP |
| rs7087591 | 10 | 92713872 | *AL590080.1, EXOC6* | GCST003400 | Regulatory site | Independent SNP |
| rs55854702 | 10 | 92716842 | *AL590080.1, EXOC6* | GCST90013693 | Intergenic | Independent SNP |
| rs565236700 | 10 | 93249423 | *RPL17P34, XRCC6P1* | GCST010118 | Intronic | Independent SNP |
| rs34872471 | 10 | 112994312 | *TCF7L2* | GCST003400 | Intronic | LD with rs7901695 |
| rs7901695 | 10 | 112994329 | *TCF7L2* | GCST010118 | Intronic | Tagged SNP |
| rs10886863 | 10 | 121169979 | *RPL19P16, LINC01153* | GCST010118 | Intergenic | Independent SNP |
| rs2106463 | 11 | 2618062 | *KCNQ1OT1, KCNQ1* | GCST90013693 | NCTEV | Tagged SNP |
| rs77402029 | 11 | 2619190 | *KCNQ1OT1, KCNQ1* | GCST007847 | NCTEV | LD with rs2106463 |
| rs2237892 | 11 | 2818521 | *KCNQ1* | GCST000219 | Intronic | Independent SNP |
| rs163182 | 11 | 2822986 | *KCNQ1* | GCST001173 | Intronic | Independent SNP |
| rs2237896 | 11 | 2837210 | *KCNQ1* | GCST003400 | Intronic | LD with rs2237897 |
| rs2237897 | 11 | 2837316 | *KCNQ1* | GCST010118 | Intronic | Tagged SNP |
| rs4148646 | 11 | 17393643 | *ABCC8* | GCST010118 | Intronic | Independent SNP |
| rs7109575 | 11 | 72752390 | *ARAP1* | GCST010118 | 5’-UTR | Independent SNP |
| rs3751236 | 12 | 27810469 | *RN7SKP15, AC009511.3* | GCST010118 | Intergenic | Independent SNP |
| rs80234489 | 12 | 31288245 | *SINHCAF* | GCST010118 | Intronic | Tagged SNP |
| rs3214606 | 12 | 31324008 | *SINHCAF* | GCST90013693 | 3’-UTR | LD with rs80234489 |
| rs118074491 | 12 | 120925703 | *RPL12P33, HNF1A-AS1* | GCST010118 | Intronic | Independent SNP |
| rs568052023 | 13 | 26207231 | *RNF6* | GCST010118 | Intronic | Independent SNP |
| rs7983505 | 13 | 32983035 | *KL, TOMM22P3* | GCST010118 | Intergenic | Independent SNP |
| rs1215468 | 13 | 80133294 | *AL137781.1* | GCST010118 | Intronic | Tagged SNP |
| rs1327315 | 13 | 80134597 | *AL137781.1, SPRY2* | GCST007847 | Intronic | LD with rs1215468 |
| rs200752045 | 13 | 80143707 | *AL137781.1, SPRY2* | GCST90013693 | Intronic | Independent SNP |
| rs9515905 | 13 | 91297308 | *MIR17HG, PPIAP23* | GCST010118 | Intergenic | Independent SNP |
| rs12907887 | 15 | 40323671 | *AC020658.2, INAFM2* | GCST010118 | Intronic | Independent SNP |
| rs4924455 | 15 | 40340937 | *CCDC9B* | GCST90013693 | Synomous | Independent SNP |
| rs8037894 | 15 | 62102065 | *NPM1P47, C2CD4B* | GCST010118 | Intergenic | Tagged SNP |
| rs7167878 | 15 | 62103990 | *NPM1P47, C2CD4B* | GCST90013693 | Intergenic | LD with rs8037894 |
| rs952472 | 15 | 77484220 | *HMG20A* | GCST010118 | 3’-UTR | Independent SNP |
| rs8026714 | 15 | 90979023 | *AC068831.8, PRC1, PRC1-AS1* | GCST010118 | Intronic | Independent SNP |
| rs117267808 | 16 | 20311846 | *GP2* | GCST010118 | Intronic | Independent SNP |
| rs1421085 | 16 | 53767042 | *FTO* | GCST010118 | Intronic | Tagged SNP |
| rs11642015 | 16 | 53768582 | *FTO* | GCST90013693 | Intronic | LD with rs1421085 |
| rs75418188 | 17 | 7042164 | *SLC16A11* | GCST90013693 | Missense | LD with rs186568031 |
| rs186568031 | 17 | 7050462 | *SLC16A11, AC120057.1* | GCST010118 | Intergenic | Tagged SNP |
| rs10908278 | 17 | 37739961 | *HNF1B* | GCST90013693 | Intronic | LD with rs8064454 |
| rs8064454 | 17 | 37741595 | *HNF1B* | GCST010118 | Intronic | Tagged SNP |
| rs11651052 | 17 | 37742390 | *HNF1B* | GCST007847 | Intronic | LD with rs8064454 |
| rs476828 | 18 | 60185354 | *AC090771.1, RNU4-17P* | GCST010118 | Intergenic | Independent SNP |
| rs142395395 | 19 | 21917904 | *ZNF43, ZNF208* | GCST010118 | Intergenic | Independent SNP |
| rs113036890 | 19 | 45654671 | *RN7SL836P, GIPR* | GCST010118 | Intergenic | Independent SNP |
| rs77300780 | 19 | 45663346 | *RN7SL836P, GIPR* | GCST007847 | Intergenic | Independent SNP |
| rs12625671 | 20 | 44366172 | *HNF4A* | GCST010118 | Intronic | Independent SNP |
| rs28691713 | 22 | 49962654 | *PIM3* | GCST010118 | Intronic | Independent SNP |

3’-UTR, 3’-untranslational region variant; 5’-UTR, 5’-untranslational region variant; NCTEV, Non-coding transcript exonic

| Table S2. Genotype distributions of 44 DM SNPs in CP-positive and –negative subjects. | | | | | | | | | | | | | |
| --- | --- | --- | --- | --- | --- | --- | --- | --- | --- | --- | --- | --- | --- |
| Candidate SNP | | Typed SNP | | Type of variant |  | Allele  A/B |  | CP-negative | |  | CP-positive | |  |
| rsid | Location | rsid | Location |  | r^2^ |  |  | A(%) | AA/AB/BB |  | A(%) | AA/AB/BB | PHWE |
| rs10634531 | 2:632790 | rs35802221 | 2:632904 | Intergenic | 1.00 | A/G |  | 7.1 | 2/58/377 |  | 6.0 | 1/35/273 | 0.89 |
| rs1260326 | 2:27508073 | rs1260326 | 2:27518370 | Intronic | 1.00 | T/C |  | 49.8 | 105/222/112 |  | 47.9 | 66/153/90 | 0.81 |
| rs11926494 | 3:23217123 | rs10510536 | 3:23228279 | Intronic | 1.00 | T/C |  | 82.7 | 296/121/14 |  | 81.3 | 202/95/10 | 0.71 |
| rs12633613 | 3:23349016 | rs12636682 | 3:23304193 | Intronic | 1.00 | A/G |  | 62.2 | 171/204/64 |  | 60.4 | 117/139/53 | 0.80 |
| rs3816157 | 3:64013646 | rs3816157 | 3:64013646 | NCTEV | 1.00 | A/G |  | 29.6 | 42/176/221 |  | 29.9 | 29/126/153 | 0.42 |
| rs13092876 | 3:185777532 | rs4402960 | 3:185793899 | Intronic | 1.00 | T/G |  | 21.8 | 20/151/268 |  | 24.8 | 18/117/174 | 0.83 |
| rs10011838 | 4:152599127 | rs17029013 | 4:152581654 | Regulatory site | 0.82 | T/C |  | 50.3 | 111/219/108 |  | 49.2 | 75/153/80 | 1.00 |
| rs256904 | 5:56514478 | rs459193 | 5:56510924 | Intergenic | 1.00 | A/G |  | 44.4 | 86/218/135 |  | 43.8 | 58/154/96 | 0.91 |
| rs4712523 | 6:20657333 | rs4712524 | 6:20657634 | Intronic | 0.96 | A/G |  | 63.3 | 178/200/61 |  | 64.6 | 122/155/32 | 0.69 |
| rs9350271 | 6:20682933 | rs2206734 | 6:20694653 | Intronic | 1.00 | T/C |  | 36.2 | 60/198/181 |  | 34.1 | 36/139/134 | 0.62 |
| rs76541615 | 6:31058459 | rs76977243 | 6:31072153 | Intergenic | 1.00 | T/C |  | 10.7 | 6/82/351 |  | 12.7 | 6/66/236 | 0.63 |
| rs2844623 | 6:31264766 | rs2844623 | 6:31264766 | Intergenic | 1.00 | T/C |  | 12.3 | 10/87/337 |  | 11.9 | 4/64/234 | 0.13 |
| rs4711389 | 6:34246893 | rs1150777 | 6:34249404 | Intronic | 0.89 | A/C |  | 88.6 | 347/82/9 |  | 86.5 | 228/77/3 | 0.12 |
| rs6918311 | 6:136966564 | rs11752908 | 6:136980861 | Intergenic | 0.94 | A/G |  | 42.4 | 81/209/147 |  | 41.6 | 52/153/104 | 0.66 |
| rs610930 | 7:69724740 | rs610930 | 7:69724740 | Intronic | 1.00 | A/G |  | 33.0 | 44/202/193 |  | 29.6 | 27/129/153 | 0.40 |
| rs12698877 | 7:70231919 | rs10256186 | 7:70176798 | Intronic | 1.00 | A/G |  | 61.8 | 165/213/61 |  | 64.1 | 122/152/35 | 0.56 |
| rs2233580 | 7:127613496 | rs3757787 | 7:127618330 | Intergenic | 0.81 | T/C |  | 11.0 | 6/84/348 |  | 13.6 | 7/70/232 | 0.72 |
| rs12549902 | 8:41651740 | rs12549902 | 8:41651740 | Intergenic | 1.00 | A/G |  | 37.7 | 61/209/169 |  | 37.2 | 44/142/123 | 0.78 |
| rs13266634 | 8:117172544 | rs13266634 | 8:117172544 | Missense | 1.00 | T/C |  | 52.4 | 127/191/107 |  | 53.0 | 95/129/77 | 0.04 |
| rs4237150 | 9:4290085 | rs7020673 | 9:4291747 | Intronic | 0.83 | CG |  | 52.1 | 118/221/100 |  | 51.3 | 83/151/75 | 0.86 |
| rs2383208 | 9:22132077 | rs2383208 | 9:22132077 | Intergenic | 1.00 | A/G |  | 59.6 | 157/209/73 |  | 56.0 | 95/156/58 | 0.81 |
| rs10965248 | 9:22132879 | rs10811661 | 9:22134095 | Intergenic | 0.98 | T/C |  | 57.6 | 143/220/76 |  | 55.0 | 91/158/60 | 0.58 |
| rs11257657 | 10:12267140 | rs11257655 | 10:12265895 | TFB | 1.00 | T/C |  | 57.5 | 146/212/80 |  | 56.5 | 103/143/63 | 0.84 |
| rs34204798 | 10:79191374 | rs12571751 | 10:79182874 | Intronic | 0.87 | A/G |  | 60.1 | 158/212/69 |  | 58.1 | 104/151/54 | 0.88 |
| rs35906730 | 10:92675916 | rs2488087 | 10:92686284 | Intergenic | 0.94 | A/G |  | 25.1 | 24/172/243 |  | 24.9 | 15/124/170 | 0.37 |
| rs7087591 | 10:92713872 | rs7923866 | 10:92722319 | Intergenic | 0.83 | T/C |  | 79.5 | 280/138/21 |  | 79.0 | 193/102/14 | 0.46 |
| rs7901695 | 10:112994329 | rs4506565 | 10:112996282 | Intronic | 1.00 | A/T |  | 97.6 | 417/21/0 |  | 98.7 | 301/8/0 | 0.61 |
| rs2106463 | 11:2618062 | rs79323575 | 11:2615006 | NCTEV | 1.00 | T/C |  | 92.5 | 373/64/1 |  | 92.6 | 264/44/1 | 0.31 |
| rs4148646 | 11:17393643 | rs757110 | 11:17396930 | Missense | 0.89 | A/C |  | 63.7 | 177/205/57 |  | 66.2 | 131/147/31 | 0.84 |
| rs7109575 | 11:72752390 | rs613937 | 11:72763794 | Intronic | 0.88 | A/G |  | 94.2 | 389/49/1 |  | 92.9 | 266/42/1 | 0.67 |
| rs3751236 | 12:27810469 | rs10842993 | 12:27811715 | Regulatory site | 0.96 | A/G |  | 39.5 | 78/190/170 |  | 36.2 | 38/148/123 | 0.05 |
| rs7983505 | 13:32983035 | rs2858980 | 13:32980449 | Intergenic | 0.97 | A/G |  | 77.9 | 262/158/18 |  | 81.6 | 202/100/7 | 0.33 |
| rs1215468 | 13:80133294 | rs1327315 | 13:80134597 | Intergenic | 0.98 | T/C |  | 29.4 | 46/166/227 |  | 30.0 | 24/137/147 | 0.06 |
| rs9515905 | 13:91297308 | rs9583907 | 13:91287016 | Intergenic | 1.00 | T/C |  | 24.1 | 27/155/252 |  | 19.6 | 14/93/202 | 0.63 |
| rs4924455 | 15:40340937 | rs1077476 | 15:40327542 | Regulatory site | 0.81 | T/G |  | 36.8 | 59/205/175 |  | 39.3 | 46/151/112 | 0.93 |
| rs952472 | 15:77484220 | rs6495240 | 15:77474214 | Intronic | 0.98 | T/C |  | 66.6 | 198/189/52 |  | 65.9 | 138/131/40 | 0.50 |
| rs8026714 | 15:90979023 | rs7180016 | 15:90983045 | Intronic | 0.93 | A/G |  | 52.4 | 115/230/94 |  | 54.5 | 98/141/70 | 0.29 |
| rs117267808 | 16:20311846 | rs4383154 | 16:20327288 | Intronic | 0.89 | A/G |  | 6.9 | 2/56/378 |  | 5.3 | 3/27/279 | 0.96 |
| rs1421085 | 16:53767042 | rs9937354 | 16:53765935 | intronic | 0.80 | A/G |  | 19.4 | 14/142/283 |  | 15.5 | 6/84/219 | 0.45 |
| rs186568031 | 17:7050462 | rs13342692 | 17:7042968 | Missense | 1.00 | T/C |  | 88.0 | 346/65/19 |  | 89.2 | 249/32/16 | <0.001 |
| rs8064454 | 17:37741595 | rs11651755 | 17:37739849 | Intronic | 0.85 | T/C |  | 72.8 | 232/175/32 |  | 74.6 | 173/115/21 | 0.90 |
| rs476828 | 18:60185354 | rs34633411 | 18:60187461 | Intergenic | 0.93 | T/C |  | 17.7 | 13/129/297 |  | 20.2 | 10/105/194 | 0.82 |
| rs12625671 | 20:44366172 | rs4812829 | 20:44360627 | Intronic | 1.00 | A/G |  | 43.8 | 84/217/138 |  | 41.4 | 46/164/99 | 0.94 |
| rs28691713 | 22:49962654 | rs137834 | 22:49988558 | Intergenic | 0.85 | A/G |  | 54.8 | 133/214/91 |  | 57.0 | 100/152/57 | 0.77 |

3’-UTR, 3’-untranslational region variant; 5’-UTR, 5’-untranslational region variant; NCTEV, Non-coding transcript exonic; TFB, transcriptional factor site variant.
